# Constitutive Aryl Hydrocarbon Receptor Signaling in Prostate Cancer Progression

**DOI:** 10.29245/2578-2967/2018/5.1136

**Published:** 2018-10-09

**Authors:** Maryam Ghotbaddini, Vivian Moultrie, Joann B. Powell

**Affiliations:** Clark Atlanta University- Center for Cancer Research and Therapeutic Development 223 James P Brawley Drive Atlanta, Georgia, USA

**Keywords:** Aryl Hydrocarbon Receptor, AR, Src Kinase, Castration Resistant Prostate Cancer, Crosstalk, Phosphorylation

## Abstract

Research on the aryl hydrocarbon receptor (AhR) has largely focused on its activation by various environmental toxins. Consequently, only limited inferences have been made regarding its constitutive activity in the absence of an exogenous ligands. Evidence has shown that AhR is constitutively active in advanced prostate cancer cell lines which model castration resistant prostate cancer (CRPC). CRPC cells can thrive in an androgen depleted environment. However, AR signaling still plays a major role. Although several mechanisms have been suggested for the sustained AR signaling, much is still unknown. Recent studies suggest that crosstalk between constitutive AhR and Src kinase may sustained AR signaling in CRPC. AhR forms a protein complex with Src and plays a role in regulating Src activity. Several groups have reported that tyrosine phosphorylation of AR protein by Src leads to AR activation, thereby promoting the development of CRPC. This review evaluates reports that implicate constitutive AhR as a key regulator of AR signaling in CRPC by utilizing Src as a signaling intermediate.

## Introduction

Most men who die of prostate cancer present with castration resistant prostate cancer (CRPC)^1^. Androgens and androgen receptor (AR) signaling play a predominant role in male sexual development, growth of the prostate gland and progression of prostate cancer to CRPC^2^. In CRPC, AR signaling has been shown to be sustained by a variety of mechanisms including increased androgen uptake by prostate cancer cells, increased AR expression, AR gene mutation and activation by other transcription factors^3–4^. The aryl hydrocarbon receptor is a transcription factor that has been extensively studied for its role in mediating the toxic effects of a wide range of environmental toxins. However, recent evidence has shown that AhR possess intrinsic functions independent of activation by an exogenous ligand. Constitutively active AhR has been shown to interact with hormone receptors and may play a role in progression of hormone-related cancers. Particularly, constitutively active AhR interacts with AR and act as a functional transcription unit^5^, a mechanism that may lead to enhanced AR signaling in CRPC.

## Constitutive AhR Signaling

Although AhR has been extensively researched for its role as a xenobiotic receptor; RNA interference, overexpression, and inhibition studies suggest a role for AhR in multiple tumor types beyond activation by environmental contaminants^6^. As an inactive complex, AhR is found in the cytosol where it interacts with tyrosine kinase c-Src as well as two molecules of HSP90, co-chaperone p23 and immunophilin-like AhR interacting protein (AIP/XAP2)^7,8^. In its active form, AhR disassociates from its chaperone proteins and dimerizes with the aryl hydrocarbon receptor nuclear translocator (ARNT). The heterodimer activates gene expression through interactions with related xenobiotic responsive elements (XREs) located on Ah- responsive gene promoters^9^. Studies concerned with the intrinsic functions of AhR have found that overexpression of the receptor may promote carcinogenesis in the absence of a exogenous ligand. AhR protein and mRNA expression is associated with phases of rapid proliferation and differentiation in certain tissues. Conversely, AhR-defective cell lines demonstrate a reduced proliferation rate^10^. Ectopic over expression of AhR in immortalized normal mammary epithelial cells induced a malignant phenotype with increased growth and acquired invasive capabilities^11^. A separate study using a constitutively active AhR construct lacking a ligand binding domain revealed that AhR acts as a transcriptional coregulator for the unliganded AR. These studies show that the endogenous AR along with the constitutively active AhR were recruited to androgen-responsive elements to initiate signaling in an androgen depleted environment^5^.

## Ligand Activated AhR Signaling

The aryl hydrocarbon locus includes AhR, ARNT, and the AhR repressor (AHRR), which are critical for regulation of AhR signaling^9^ in both constitutive and ligand activated signaling. AhR ligands such as 2,3,7,8-tetrachlorodibenzo- p-dioxin (TCDD), components of cigarette smoke such as benzo(a)pyrene (BaP), and a wide range of polycyclic aromatic hydrocarbons (PAH) exert their biological influences by binding directly to the AhR. Following ligand activation, AhR’s primary role is identified as the control of xenobiotic metabolism through cytochrome P450s, some of which are transcriptional targets of AhR^13^. Activation of AhR by PAHs has been reported to antagonize AR signaling. For example, TCDD has been shown to alter sex steroid hormone secretions. TCDD was also shown to block the androgen dependent proliferation of prostate cancer cells^14^. Simultaneous activation of AhR and AR with TCDD and an androgen derivative, respectively, decreased AR protein levels^15^. Thus, activation of AhR by a ligand results in decreased protein expression of both AhR and AR. This action may explain the anti- androgenic actions of a number of PAHs, is distinctive from the effects seen with constitutive AhR signaling that accompanies overexpression of the AhR protein and may be the result of enhanced activity of AhR chaperone protein, Src kinase.

**Figure 1:**
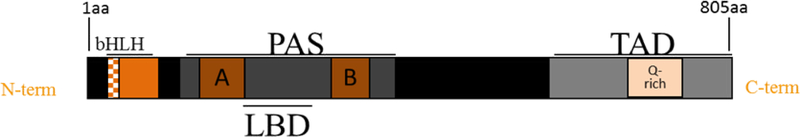
The schematic structure of AhR functional domains. The AhR protein contains several domains critical for transcriptional activity. The basic-helix-loop-helix (bHLH) motif, two Per- Arnt-Sim (PAS) domains (PAS-A and PAS-B) and a glutamine rich transactivation domain (TAD).

The mammalian AhR protein contains four major structural motifs important in AhR’s interactions with other proteins and transcription factors. The N-terminal basic-helix-loop-helix is the site of DNA binding domain and also participates in dimerization and HSP90 binding. The transactivation domain spans from amino acid 490 to 805 and includes a central glutamine rich region. The two Per-Arnt-Sim (PAS) domains are named after their homology with the clock protein period (Per), the xenobiotic and oxygen sensing ARNT, and the neuronal cell lineage regulator single-minded (Sim) (Figure 1)^16^. The PAS domains facilitate interactions with other PAS domain proteins, such as the AhR binding partner, ARNT PAS-A is primarily responsible for protein-protein interaction and PAS-B also encompasses the ligand binding^17^. cSrc may interact directly with the AhR transactivation and PAS domains^7^. Western blot and FRET analysis confirm time- dependent phosphorylation of Src following activation of AhR which was blocked in the presence of a specific AhR antagonist^18^. Furthermore, coimmunopercipitation experiments revealed that AhR regulates Src activity by phosphorylating Src (Tyr 416) and dephosphorylating Src (Tyr527)^19^.

## Src activity in prostate cancer

The increased expression of Src and other Src family kinases (SFK) in a number of prostate cancer cell lines has suggested a role for Src in prostate cancer initiation and progression^20^. There are also many reports that SFKs are abnormally activated in prostate cancer cells. SFKs are activated in response to numerous stimuli including neuroendocrine ligands, reactive oxygen species, cytokines and growth factors. These molecules have proven roles in cancer progression, including cell proliferation, adhesion, migration, and invasion^21^.

Src and AhR coexist in a protein complex that also contains HSP90, AhR-interacting protein, and p23 that aides to maintain an inactive AhR complex^8^. TCDD activation of AhR results in time- and dose-dependent phosphorylation of Src (Tyr416). Reported Src-mediated crosstalk between AhR and EGFR signaling pathways demonstrated an important link between AhR canonical function and TCDD-mediated tumor promotion^19,22,23^. Through direct physical interaction with AR, Src is able to phosphorylate AR and thereby induce ligand-independent activation of AR, a key mechanisms of CRPC^24^. Conversely, AR overexpression also plays a role in the oncogenic potential of wild-type Src. This suggests that crosstalk and activation of AR and Src is reciprocal^25^.

## Tyrosine Phosphorylation of AR

AR contains at least sixteen phosphorylated residues. Several of the residues are phosphorylated after treatment of cells with androgen, antiandrogen, or reagents which activate other signaling pathways and alter transcriptional activity, cellular localization, and stability of AR^26^. As a phosphoprotein, AR has several serine/ threonine and tyrosine residues that are phosphorylated. Most of the phosphorylated residues are located in N-terminal domain that regulates AR cellular localization, stability and its transcriptional activity.

**Figure 2:**
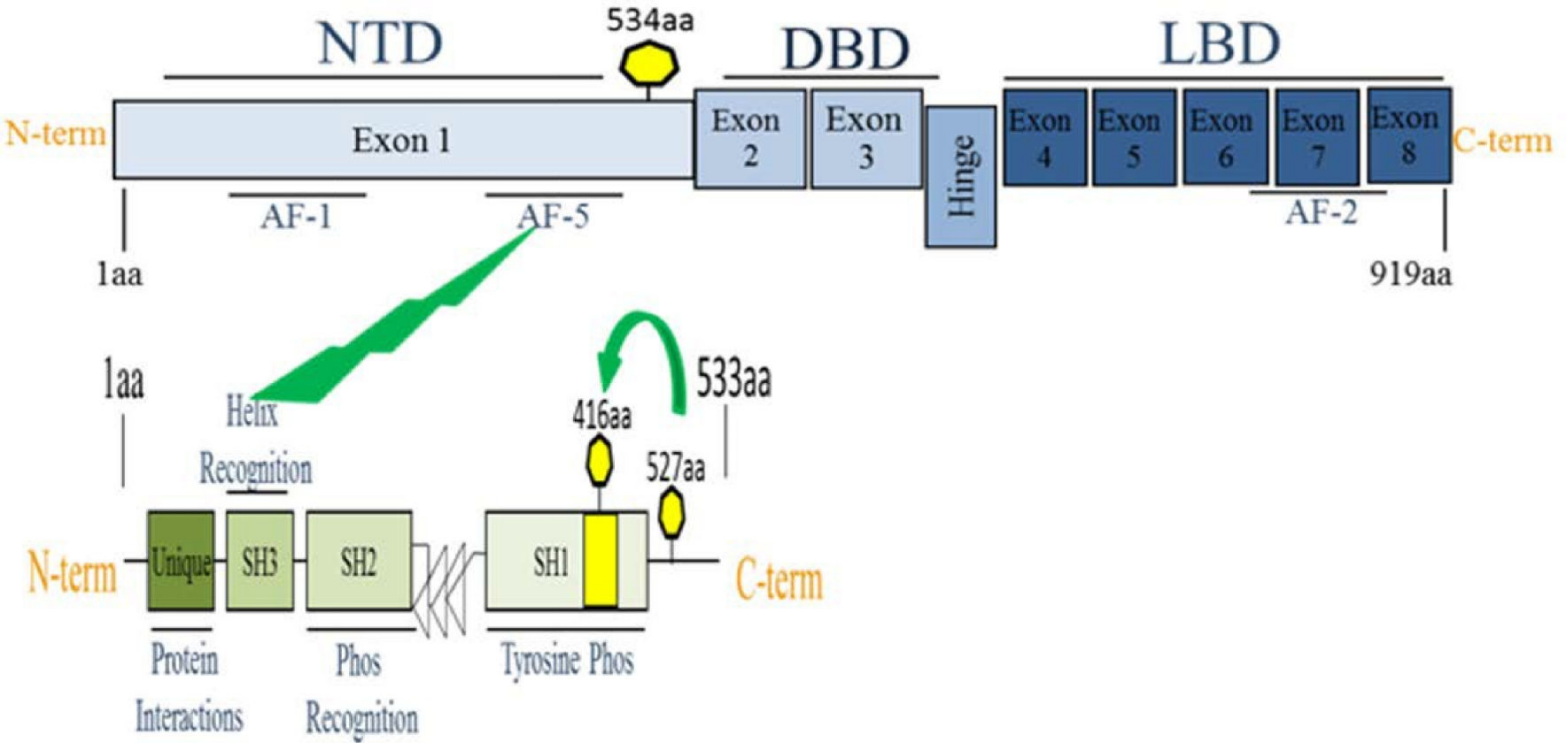
Schematic structure of Src and AhR interaction and phosphorylation. The AR protein contain an N-terminal domain (NTD); DNA-binding domain (DBD); and ligand-binding domain (LBD). AF-1, AF-5, AF-2 are three known transactivation domains in the AR. The Src molecule AR interacts with the AF-5 domain of AR through the SH3 (helix recognition) domain of cSrc. This interaction occurs following phosphorylation of Tyr416 and dephosphorylating of Tyr527 which is located in a C-terminal regulatory domain.

Recently, several groups have reported that tyrosine phosphorylation of AR protein by non- receptor tyrosine kinases Src may have a role in AR activation in the low androgen environment, thereby promoting the development of CRPC. Src-mediated phosphorylation of AR at Y534 resulted in the activation of AR followed by nuclear translocation and DNA binding in the absence of androgens (Figure 2)^26–28^.

## AR signaling and regulation prostate cancer

AR is a member of the steroid hormone receptor family and shares a similar domain organization with other members of the nuclear receptor (NR) which is primarily responsible for mediating the physiological effects of androgens by binding to AREs^29^. In the presence of low levels of androgen, AR is normally localized to the cytosol in a complex with molecular chaperones, Hsp40, 70 and 90 in an inactive form. Upon androgen binding, the androgen induces conformational changes in the protein, forms a homodimer, and translocates to the nucleus^30^. The nuclear translocation results in binding of AR as a transcription factor to ARE’s in the regulatory region of target genes. AR signaling plays a critical role in prostate cancer cell proliferation, survival, and differentiation^31^.

Several studies based on molecular cloning of AR cDNA, suggest that its transcriptional activity is critical for all stages of prostate cancer development and progression. Several neutral next- generation sequencing platforms have been used to pursue genomic characterization of prostate cancer at various stages. Results have consistently confirmed a critical role for AR activity in prostate cancer progression^32^.

While AR signaling is known to modulate the expression of genes associated with cell cycle regulation, survival and growth, its specific functions are not fully defined in prostate cancer^33^. Whole genome sequencing of 11 early onset prostate cancers suggested that androgens, through AR, contribute in shaping somatic alterations^34^. These results are further supported by studies demonstrating that AR is known to stimulate the expression of TMPRSS2: ERG which is a common gene fusion associated with prostate cancer initiation via androgen-driven overexpression of the gene fusion products^35^. It is clear that AR signaling plays a critical role in the development and progression of prostate cancer and serves as a central component of progression to CRPC.

## Crosstalk between AhR and AR signaling

AhR signaling influences androgen signaling directly at the level of the protein and indirectly through actions on the endocrine system. AhR plays a significant role in sustained AR signaling and growth. However, the mechanism for this role is not clearly understood. One possible mechanism is direct interaction between AhR with AR. AhR has been reported to directly interact with a number of nuclear proteins^36–38^. Direct heterodimerization of AhR and AR has been shown to occur in cells and may partially explain the crosstalk between the two receptors^5^. Additionally, interaction can happen via coactivators. AhR and AR share a number of coactivator proteins such as SRC1 and p300^39^. Another possible mechanism AhR may utilize for AR activation is phosphorylation of AR by Src kinase^5,40^. Src was shown to mediate crosstalk between AhR and epidermal growth factor receptor in colon cancer cells^19^. Other studies have shown that Src kinase can promote AR transactivation in C4–2 cells prostate cancer cells. Consequently, inhibition of Src kinase function with a specific inhibitor resulted in decreased AR activation^41^. Coimmunoprecipitation experiments revealed that AhR forms a protein complex with Src and regulates Src activity by phosphorylating Src (Tyr416) and dephosphorylating Src (Tyr527)^19,42^. Immunoprecipitation assays also revealed the association of AR with Src, suggesting complex formation among them^43^.

## Conclusion

The precise molecular mechanism utilized by constitutive AhR signaling to activate AR signaling needs to be investigated further and could include induced activation via protein phosphorylation, direct heterodimerization and interacting via coactivators. Extensive *in vitro* and *in vivo* studies have established a role for AhR in prostate and prostate cancer development. The development of AhR-null mice has revealed that the function of this receptor is not limited to mediating the effects of PAHs and other ligands^44^. AhR knockout mice exhibit decreased fertility, decreased liver size, and structural and functional deficits in several tissues^45^. These include reproductive tract problems such as decreased levels of mature follicles and formation of uric acid stones in the urinary bladder^46^.

**Figure 3:**
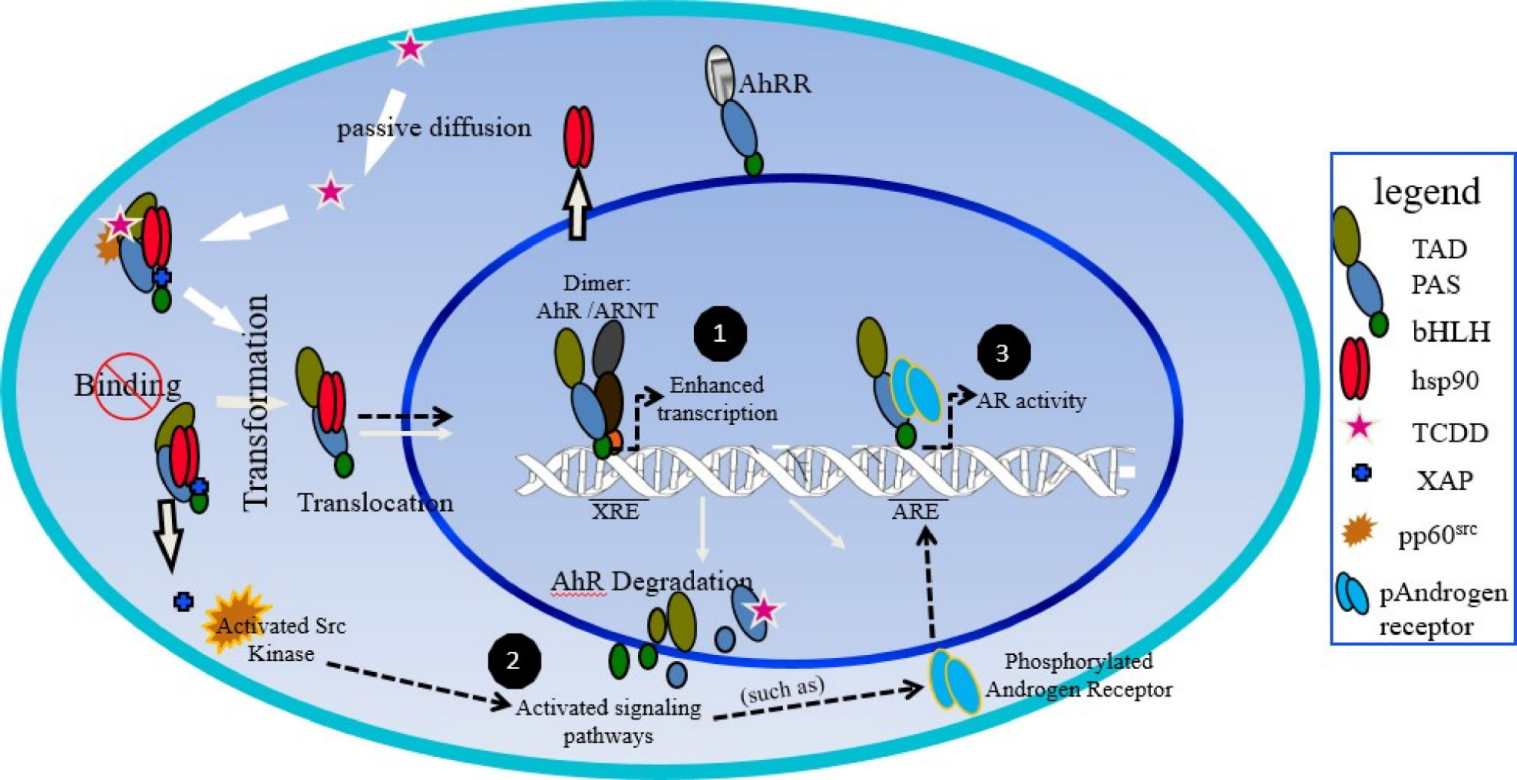
The proposed pathway for constitutive AhR signaling and crosstalk with Src. (1) Binding of various exogenous and endogenous ligands to the cytoplasmic AhR or deletion of the ligand binding domain stimulates translocation to the nucleus where the AhR/ARNT heterodimer forms. The AhR-ARNT dimer binds to a cognate xenobiotic response element (XRE) to induce transcription of genes important in a wide range of biological processes. (2). AhR forms aprotein complex with Src and regulates Src activity by phosphorylating Src at Tyr416 and dephosphorylating Src at Tyr527. (3) Phosphorylation of AR protein by non-receptor tyrosine kinases Src.

AhR and ARNT are expressed through all stages of male urogenital system development and in the prostate gland. AhR and ARNT genes are expressed in fetal mouse and rat urogenital sinus (UGS) and in normal, hyperplastic, and cancerous adult human prostate tissue^47^. Ligand activation of AhR by TCDD is adequate to disrupt key stages of ductal morphogenesis. Significantly smaller prostate lobes were observed in rats and mice exposed to TCDD during fetalpubertal development. Indicating that TCDD-induced AhR activation delays prostate growth^48^. TCDD exposure also led to a decreased number of prostate ducts in monkeys^49^. AhR may serve as a key regulator of AR signaling in CRPC by utilizing Src as a signaling intermediate. Recent studies have revealed that simultaneous inhibition of AhR and Src is sufficient to abolish AR signaling in CRPC cells^50^. Activated Src is a common signaling intermediate in a number of pathways including AR signaling pathway and has increased activity in CRPC. Although several mechanisms have been suggested for the sustained AR signaling, tyrosine phosphorylation of AR protein by AhR- activated-Src may have a significant role in CRPC that also exhibit constitutive AhR signaling (Figure 3).
